# Implementation of a patient safety training program in radiation oncology residency: A pilot study

**DOI:** 10.1002/acm2.14286

**Published:** 2024-02-15

**Authors:** Imran H. Chowdhury, Rashi Garg, Kathryn E. Huber, Nathaniel P. Stambaugh, Cassandra Stambaugh

**Affiliations:** ^1^ Department of Radiation Oncology Tufts University School of Medicine Boston Massachusetts USA; ^2^ Department of Radiation Oncology Oncology Hematology Care, Inc. Cincinnati Ohio USA; ^3^ Department of Radiation Oncology Perelman School of Medicine University of Pennsylvania Philadelphia Pennsylvania USA; ^4^ Department of Math and Science Southfield School, Dexter Brookline Massachusetts USA

**Keywords:** education, RO‐ILS, safety training, training program

## Abstract

**Purpose:**

An educational program using Radiation Oncology‐Incident Learning System (RO‐ILS) was developed to improve safety culture and training for radiation oncology (RO) residents.

**Methods:**

The program included a pre‐training assessment, interactive training, integration of residents into quality assurance meetings, and a post‐training assessment over a 3 month rotation.

**Results:**

Twelve residents completed the safety training program. Pre‐training assessment mean scores (five‐point scale) of experience with Incident Learning Systems (ILS), root‐cause analysis (RCA), failure‐mode and effect analysis (FMEA), safety training, and culture were 2.3, 2.8, 2.0, 4.0, and 4.4, respectively. Post‐training assessment showed a significant increase in ILS 4.0 (*p *< 0.001), RCA 3.8 (*p *= 0.008), and FMEA 3.3 (*p *= 0.006) and safety culture (4.8, *p *= 0.043). Additionally, residents were anonymously surveyed ≥ 10 months after graduation to determine the long‐term value of the program. The overall assessment from the graduated residents indicates that this education is valued by RO in many institutions. The majority of the residents are either currently utilizing or plan to utilize the information gained in this program in their new institutions.

**Conclusions:**

We report a successful implementation of a safety training program in a RO residency with significant improvements in self‐reported confidence with the concepts of ILS, RCA, and FMEA and an improved perception of safety culture. This program can be implemented across all residency programs.

## 1 INTRODUCTION AND INNOVATION

Patient safety and quality improvement (QI) have been a significant focus in Radiation Oncology (RO) over the past decade.^1^ A positive culture of safety within the clinic is associated with fewer adverse events and improves overall patient safety and quality.^2^, ^3^ Incident learning has been recognized as a critical tool to help mitigate errors and improve the quality of care.^1^, ^4^ It has been shown that increased reporting of events has been associated with lower rates of negative patient safety indicators.^2^, ^3^ As a part of the American Society of Radiation Oncology's (ASTRO) “Target Safely” initiative, ASTRO and the American Association of Physicists in Medicine (AAPM) have implemented radiation oncology incident learning system (RO‐ILS), a national specialty‐specific, Incident Learning System (ILS) to help improve patient safety and quality in RO clinics.^5^

While medical physicists often implement and lead QI projects and programs, radiation oncologists are expected to help implement and promote a positive culture of safety in the clinic. The Accreditation Council for Graduate Medical Education (ACGME) has given general guidelines for patient safety training in residency programs. However, most institutions implement these guidelines as web‐based modules during residency onboarding with no continuing education.^6^ A 2017 survey revealed that most RO and medical physics residents had minimal‐to‐no exposure to standard incident learning tools and that a minority of residents felt that their safety training was adequate.^7^ Therefore, we developed a safety training program for RO residents utilizing incident learning led by a medical physicist. This pilot study highlights the outcomes of implementing such a training program.

### 1.1 Training program

Our department is the sponsoring institution of a RO residency with nine residents who rotate to three institutions. Each rotation is 3 months, and residents may return to the sponsoring institution for multiple rotations throughout their residency. RO residents only participate in RO‐ILS while at the sponsoring institution.

RO‐ILS was implemented in our clinic in 2019. In the fall of 2019, our institution officially implemented a formal safety training and knowledge self‐assessment program using RO‐ILS as the primary tool in the RO residency curriculum. Residents were added as reviewers in the RO‐ILS system during the training program. A beta feature within the portal at the time, “Task Manager,” was enabled, allowing events to be assigned to residents to review and complete pre‐set follow‐up questions designed to provide additional information on the event, such as identifying the problem type, when the event was discovered versus when it occurred, and the impact on the patient. In the same system they indicate contributing factors of RO‐ILS events.

The training first consisted of a 10‐question pre‐training self‐assessment (Supplemental File S1) at the beginning of the residents' rotation at the sponsoring institution. The first five questions evaluated the residents' self‐assessed experience level with ILS, root‐cause analysis (RCA), failure modes and effect analysis (FMEA), and their opinion regarding safety culture and training at our institution. These questions were assessed using a 5‐point Likert scale from 1 – very weak to 5 – very strong. The final five questions were multiple‐choice or true/false questions scored as pass/fail and tested specific concepts of patient safety and QI to be covered in the training lecture. These questions were based on the training material provided by ASTRO for RO‐ILS.

**Learning Objectives**

1. To understand concepts of safety culture, root cause analysis, and failure mode and effects analysis, and how it relates to patient safety
2. To demonstrate learning transfer by completing five incident learning follow‐ups
3. To develop confidence in the incident learning process

After the pre‐training self‐assessment, a 1‐hour lecture (Supplemental file S2) was given to the residents by a board‐certified medical physicist, which consisted of background on RO‐ILS (adapted from documentation provided by ASTRO for clinical training for RO‐ILS) and the submission and follow‐up process of submitted events. Follow‐ups required residents to interview relevant staff/groups to learn more about the event and to gather the necessary information to fill out the pre‐set follow‐up questions. Therefore, the lecture included instructions for interacting with event reporters and staff, such as: scheduling time, not interrupting other tasks, and providing a summary of the event to allow the staff member to be prepared. Additionally, residents were tasked with identifying contributing factors of the event, which required the resident to perform an informal, mini‐RCA. Therefore, an interactive introduction to RCA and FMEA was included as part of the lecture and during supervised follow‐ups. This included going through the process steps for each analysis method using sample events that the residents brainstorm during the lecture period. While it is not possible to perform a full RCA or FMEA in a lecture period, a simplified analysis allows the residents to see the pros, cons and limitations for each method. RCA was further emphasized to ensure residents understood how to effectively determine contributing factors.

RO‐ILS events were then assigned to residents, with the first follow‐up done under the supervision of a trained attending faculty. During supervised follow‐ups, when appropriate, questions would be asked of the residents to further discuss the differences between RCA and FMEA and whether an FMEA on that workflow would have prevented the reported event. This reinforced the different methodologies as well as the advantage of the FMEA process in preventing future problems by looking ahead at what errors could occur. After completing the first supervised follow‐up, the rotating residents were integrated into the regular rotation of RO‐ILS follow‐ups. They were expected to complete at least five follow‐ups independently. Each RO‐ILS event and follow‐up were reviewed during our bi‐monthly QA meeting. During the meeting, feedback was provided to the resident if any modifications were needed. At the end of the clinical rotation, the residents took a post‐training self‐assessment. Training and self‐assessments were repeated if a resident returned to the sponsoring institution during the study period to evaluate the impact on reported experience and comfort level with the material during a break in hands‐on training and after repeat training.

### 1.2 Longitudinal follow‐up

An anonymous post‐residency survey (Supplemental file S3) was sent to the residents who had graduated from our program at least 10 months after graduation. The survey consisted of six background questions (yes/no/maybe responses) and five questions specific to the incident learning program. These questions were assessed using a four‐point Likert scale ranging from 1 – not valuable/not at all/ not very likely to 4 – very valuable/very much/very likely. This survey assessed the value of the incident learning education program on board preparation, their current role, general knowledge, if they have used the knowledge gained from the program, and if they think they will utilize it in the future.

### 1.3 Training results

A total of 12 residents completed the safety training program, with five completing it at least twice with a minimum span of 6 months between training sessions. Each resident training session was 1 hour for the lecture and 30 min per resident to supervise the initial incident follow‐up report. Each participating resident completed five follow‐ups during their rotation (∼15 min per follow‐up). Pre‐training self‐assessment results of the training residents' self‐reported experience levels in ILS, RCA, and FMEA had mean scores (five‐point scale) of 2.3, 2.8, and 2.0 (Table 1). Scores for safety training and culture were 4.0 and 4.4, respectively. Post‐training self‐assessment results showed a significant increase in the sample mean experience level scores. The rating in ILS showed an increase to 4.0 (*p *< 0.001), RCA increased to 3.8 (*p *= 0.008), and FMEA increased to 3.3 (*p *= 0.006). The safety training rating was unchanged for most residents, so the average increase to 4.3 was not statistically significant (*p* = 0.203). Their rating of safety culture also did not change for most residents, but every change was positive so the increase to 4.8 was statistically significant (*p *= 0.043). For the residents that participated in a second training session, the second pre‐training self‐assessment showed regression or no improvement in the reported experience level of ILS, RCA, and FMEA compared to the post‐training survey of the first training session (see Table 1 post‐training #1 to pre‐training #2). However, after completing the second training session, the post‐training self‐assessment showed reported experience levels at or exceeding the post‐training survey of the first training session. The knowledge‐based questions were scored out of five points, and pre‐training results scores averaged 4.3, while post‐training, they were 4.5, which was not a significant increase.

**TABLE 1:** Pre‐training and post‐training sample mean results for each analyzed category.

First training session: Sample means for entire group 1 – very weak to 5 – very strong.					
	Incident learning	Root cause analysis	FMEA	Safety training	Safety culture
Pre‐training mean [standard error] (*n* = 12)	2.3 [0.3]	2.8 [0.2]	2.0 [0.3]	4.0 [0.2]	4.4 [0.2]
Post‐training mean [standard error] (*n* = 12)	4.0 [0.3]	3.8 [0.4]	3.3 [0.3]	4.3 [0.2]	4.8 [0.1]

Repeat Training session: sample means for group who had two training sessions					
Pre‐training #1 mean [standard error] (*n* = 5)	2.0 [0.4]	2.6 [0.2]	1.6 [0.2]	3.8 [0.4]	4.0 [0.0]
Post‐training #1 mean [standard error] (*n* = 5)	3.8 [0.2]	3.4 [0.4]	2.8 [0.4]	4.0 [0.0]	4.8 [0.2]
Pre‐training #2 mean [standard error] (*n* = 5)	3.4 [0.2]	2.8 [0.4]	2.4 [0.2]	3.6 [0.2]	4.2 [0.4]
Post‐training #2 mean [standard error] (*n* = 5)	4.0 [0.0]	3.4 [0.2]	3.0 [0.3]	4.6 [0.2]	4.8 [0.2]

Abbreviation: FMEA, failure‐mode and effect analysis.

The 10 most recent resident graduates were surveyed. Nine responses were received (response rate 90%). Of those, seven indicated completing the incident learning education program and two did not rotate to the sponsoring institution during the study time‐period. Of the respondents, 55% (5/9) are at institutions with incident learning programs, and all but one of those programs is specific to RO. Of the ones that do not (4/9), when asked if they had plans to help implement a program in the future, two former residents indicated that “I would like to, but I do not have the time resources to dedicate to this” and one indicated “Yes, and I feel that I have the time, resources, and support to do so” and indicated that they were actively working on the implementation. Three out of seven residents found this program “valuable,” three found it “somewhat valuable,” and one found it “not valuable” to board preparation. The majority of residents found this program “very valuable” (3/7) or “valuable” (1/7) to their current role, and the rest (3/7) found it “somewhat valuable.” The majority (3/7) also found the program “very valuable” to their general knowledge, while three out of seven found it “valuable,” and one found it “somewhat valuable.” All residents found the program valuable ( ≥ 3) in at least one category. Two of the seven have “very much” utilized the knowledge gained post‐residency, while all but one had used the knowledge gained in the program in some capacity (2/7 “used,” 2/7 “somewhat used”). All anticipate utilizing the knowledge at some point (3/7 “very likely,” 2/7 “likely,” 2/7 “somewhat likely”). The overall assessment of the incident learning program was positive and is summarized in Table 2.

**TABLE 2:** Post residency assessment of incident learning program.

Post residency incident learning program assessment 1 – not valuable/not at all/ not very likely to 4 –very valuable/very much/very likely					
	How valuable was the RO‐ILS education program to your board preparation?	How valuable was the RO‐ILS education program to your current role?	How valuable do you feel the RO‐ILS education program was to your general knowledge?	Have you utilized your RO‐ILS education program knowledge post residency?	How likely are you to use your patient safety knowledge gained by the RO‐ILS education program in the future?
Mean [standard error] (*n* = 7)	2.3 [0.3]	3.0 [0.4]	3.3 [0.3]	2.7 [0.4]	3.1 [0.3]

Abbreviation: RO‐ILS, radiation oncology‐incident learning system.

### 1.4 Statistical analysis

Data analysis was performed using R (R Development Core Team, 2021). Repeated measurements were analyzed using a non‐parametric test (Wilcoxon rank sum test with continuity corrections) as the change in their responses to Likert scale questions failed the Shapiro‐Wilk normality test.

## 2 DISCUSSION

The impetus for this pilot study was to build on the work of Spraker et al., who surveyed 195 medical and physics RO residents and demonstrated inconsistent quality and content of patient safety training.^7^ Over 60% of residents reported having no or only informal exposure to ILS, RCA, and FMEA, and 73% felt that their safety training was inadequate. Residents preferred having more hands‐on safety training. The above contrasts a survey given to RO and medical physics program directors who reported that residents had sufficient patient safety/QI training and were qualified to meet expectations in practice.^8^ Others have reported on a safety training program previously developed for medical physics residents, which consisted of readings, video‐based lectures, and participation in a safety/QI project and departmental ILS.^6^ They report the results and experience of five residents who completed this program with an overall positive experience.

In this pilot study, we describe the implementation of a patient safety training program using RO‐ILS as the primary ILS tool in the regular curriculum of a RO residency. Among the 12 residents who participated, there was a significant increase in their self‐reported experience level and comfort in the concepts of ILS, FMEA, and RCA. The safety training program also significantly increased the residents' perception of the safety culture at our institution. The increase in perception of safety training was not statistically significant, suggesting an initial higher rating in anticipation of their training. We also observed a lack of improvement, or regression, in residents' self‐reported experience and comfort level after the initial training program when not actively participating in a safety training program. After a second training, the residents' reported experience and comfort with ILS, RCA, and FMEA were stronger than the initial training and persisted through subsequent training sessions. The lack of improvement, or regression, indicates that active participation in a hands‐on incident learning training program correlates with an increase in reported experience and comfort level with safety culture and incident learning, while it is difficult for residents to gain these benefits in departments that do not actively engage residents in these processes. Additionally, this suggests that programs that have a one‐time training may report sufficient training, but the residents may report inadequate training due to the atrophy of experience post‐training.

Using a small number of multiple‐choice and true/false questions to assess knowledge proved challenging to measure significance. Open responses or a larger number of assessment questions may be more appropriate for knowledge assessment. The lasting impact of this program was assessed by anonymously surveying residents at least 10 months post‐residency. The overall assessment from the graduated residents indicates that radiation oncologists value this education in many institutions. Most residents are either currently utilizing or plan to utilize the information gained in this program for the roles in their new institutions. Providing safety culture and incident learning education during RO residency allows residents to enter roles after graduation with an excellent foundational knowledge of these topics and transition to actively contributing to or implementing their own programs. This is indicated by the resident graduates when asked about the program:

“RO‐ILS is very common, and familiarity with this system is helpful in most post‐residency employment settings. The foundational principles of practice improvement are also quite valuable.”

“Currently working on implementing ROILS in my current practice. I could not have pursued this without my fundamental knowledge of ILS and ROILS that I learned [in this program].”

To our knowledge, this study is the first to report the successful implementation of a hands‐on safety training program for RO residents, who traditionally are less involved with QI and ILS, using the RO‐ILS platform. Using RO‐ILS as the platform for this training was beneficial and served multiple needs. Residents received hands‐on training on investigating an event, which gave them a better understanding of underlying safety concerns and why medical errors occur. In addition, resident participation also helped alleviate some review workload from other staff. The time spent on the training lecture was offset by the reduction in events needing follow‐up for staff. Since each report takes ∼15 min, on average the time cost for training was 15 min to aid in the initial follow‐up report. There was an overall time‐saving if the residents were grouped for the lecture. The lasting impact demonstrated by this program from the longitudinal survey is a powerful indicator of the importance of incorporating hands‐on safety training in RO residencies throughout their training. By incorporating the RO resident training into a clinical workflow, the impact on resources for on‐going training can be minimized.

There are some limitations to this study. Not all residency programs have an established ILS, such as RO‐ILS. However, our program can be modified to any home‐grown RO event learning system. Also, as mentioned previously, the methodology for assessing knowledge gained was not sufficient for measuring significant difference in competence pre to post training in this cohort. More work would need to be done to quantitatively measure competence versus confidence gained. However, all residents in this study were able to take the knowledge gained from lecture material and hands‐on training and apply it to perform event follow‐ups independently.

## 3 CONCLUSION

In this study, we report the successful implementation and lasting impact of a safety training program in a RO residency with significant improvements in self‐assessed experience and confidence in ILS, RCA, and FMEA concepts. This program can be implemented across all residency programs. The training program also improves residents' perception of safety culture. Continued work and a multifaceted approach are needed to help fill the gaps in safety training education and improve the organizational safety culture within RO clinics.

## AUTHOR CONTRIBUTIONS

Cassandra Stambaugh, Kathryn Huber, and Rashi Garg designed and implemented program; Imran Chowdhury aided in the data collection and writing; Nathaniel Stambaugh did the statistical analysis. All authors contributed to the writing and editing of the manuscript.

## CONFLICT OF INTEREST STATEMENT

The authors declare no conflicts of interest.

## DATA AVAILABILITY SHARING

Research data are stored in an institutional repository and will be shared upon request to the corresponding author.

## Supporting information

Supporting Information

Supporting Information

Supporting Information
