# Supplementary material for: Implementation of a patient safety training program in radiation oncology residency: A pilot study
Source: J Appl Clin Med Phys. 2024 Feb 15;25(3):e14286. doi: 10.1002/acm2.14286 (PMC10929992; doi:10.1002/acm2.14286)
Supplement: Supplementary file 1 — Supporting Information [file ACM2-25-e14286-s003.docx]

Supplemental 1

**Safety/RO-ILS Resident Self-Assessment**

Please rate the following based on your experience

1. How would you rate your experience level with an incident learning system?
   1. Very weak
   2. Weak
   3. Moderate
   4. Strong
   5. Very strong
2. What is your experience level with root cause analysis?
   1. Very weak
   2. Weak
   3. Moderate
   4. Strong
   5. Very strong
3. What is your experience level of an FMEA?
   1. Very weak
   2. Weak
   3. Moderate
   4. Strong
   5. Very strong
4. How would you rate the safety training in your residency program?
   1. Very weak
   2. Weak
   3. Moderate
   4. Strong
   5. Very strong
5. How would you rate the safety culture at your institution?
   1. Very weak
   2. Weak
   3. Moderate
   4. Strong
   5. Very strong

Please answer the following to the best of your ability

1. A safety culture includes all of the following except:
   1. attitudes, beliefs, perceptions, and values that employees share in relation to safety
   2. backwards accountability
   3. error disclosure to the patient
   4. debriefing programs or stress management programs to help practitioners after incidents
2. Which of these correctly describes care delivery problems?
   1. Care within the safe limits of practice
   2. Actions or omissions by staff which leads to an eventual adverse outcome for patients or staff
   3. Consequences that arise after there has been deviation beyond the safe limits of practice
3. A root cause analysis can be performed on both near misses and incidents
   1. True
   2. False
4. Changes that can occur with the use of an incident learning system such as RO-ILS include all of the following except:
   1. Improved communication
   2. Changes to existing policies/workflow
   3. Increase monitoring of problem areas
   4. Allocation of resources/staff
   5. Create a punitive work environment
5. Failure modes and effects analysis is a step by step approach for assessing postulated failure modes in a clinical process
   1. True
   2. False
