# Supplementary material for: Implementation of a patient safety training program in radiation oncology residency: A pilot study
Source: J Appl Clin Med Phys. 2024 Feb 15;25(3):e14286. doi: 10.1002/acm2.14286 (PMC10929992; doi:10.1002/acm2.14286)
Supplement: Supplementary file 2 — Supporting Information [file ACM2-25-e14286-s001.pdf]

Educational Goals

- Enhance the learner's understanding of safety culture and how it relates to patient safety
- Introduction to our incident learning system, RO-ILS and familiarization with steps on how to submit an event
- Understanding the key role of a root cause analysis in the follow up process
- Explaining failure mode and effects analysis and its importance in identifying deficiencies in a process

Safety Culture

- The attitudes, beliefs, perceptions, and values that employees share in relation to safety
- An organization has to :
  - Want to improve
  - Be self aware enough to improve
  - Have the resources to improve (ie- an incident learning system)
  - Be empowered to make changes
  - Get leadership support to improve
- Forward accountability is change oriented vs backwards is focused on retribution
- A robust safe culture includes error disclosure to the patient

Safety Culture

- How does a safety culture treat error?
  - An incident must not be seen as a failure or crisis , neither by management, nor by colleagues.
  - An incident is a free lesson, a great opportunity to focus attention and to learn collectively.
  - Support for the "second victim"
  - Safety cultures implement and evaluate debriefing programs or critical incident/stress management programs the organization may have in place to help practitioners after incidents
    - Practitioners must see that incidents are normal
    - Incidents can help the organization get better
    - Incidents can and DO happen to everybody
      - -Dekkar, Just Culture

Safety Culture: Who investigates?

- The direct manager of the practitioner should not necessarily be the one who is the first to handle the practitioner in the wake of an incident
- Decouple the incident from any hint of performance review or punishment
    - -Dekkar, Just Culture

Care Delivery Problems aka "Unsafe Acts"

- CDPs are problems that arise in the process of care, usually actions or omissions by members of staff
- Several CDPs may be involved in one incident
- They have two essential features:
  - Care deviation beyond safe limits of practice
  - The deviation had at least a potential direct or indirect effect on the eventual adverse outcome for the patient, member or staff or general public
- Examples:
  - Failure to monitor, observe, or act
  - Incorrect (with hindsight) decision
  - Not seeking help with necessary

Contributing Factors to CDP

- Patient factors
- Task and technology factors
- Individual factors
- Team factors
- Work environment factors

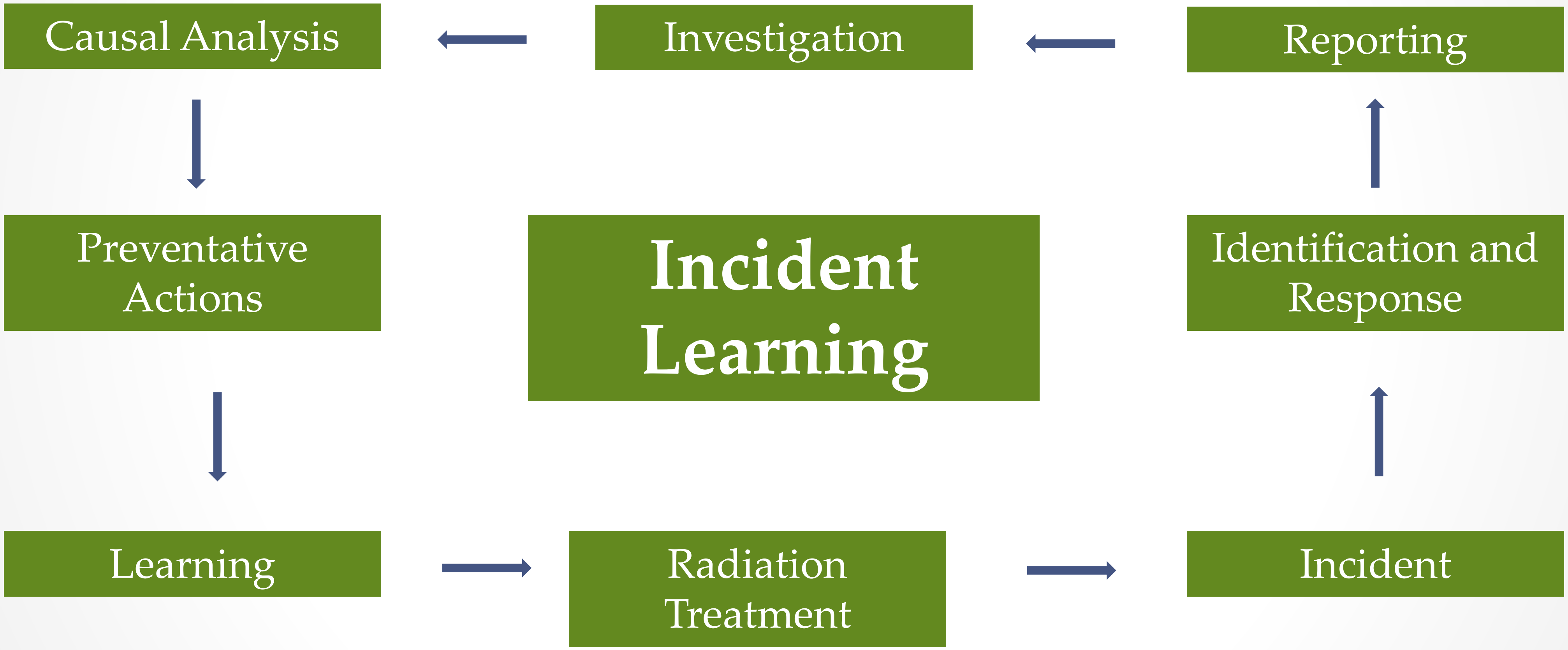
Causal Analysis
Investigation
Reporting
Preventative Actions
Incident Learning
Identification and Response
Learning
Radiation Treatment
Incident

RO-ILS BACKGROUND AND OVERVIEW

Adapted from the RO-ILS Implementation Powerpoint

Background

- *Patient Safety and Quality Improvement Act of 2005 (PSQIA)*

Congress recognized the need to collect patient safety data in a protected space. PSQIA authorized the following:

- *Patient Safety Organizations (PSOs)* – entity with expertise in patient safety authorized to work with providers under the protections of PSQIA.
- *Patient Safety Evaluation System (PSES)* – the collection, management, or analysis of information for the purposes of quality and safety improvement and for reporting to or by a PSO. Protected space where PSWP is produced and resides.
- *Patient Safety Work Product (PSWP)* – Information that is privileged and confidential under the PSQIA:

What is RO-ILS?

- RO-ILS is an online, safety data collection instrument tied with a federally listed patient safety organization (PSO), Clarity PSO.
- Clarity PSO provides associated PSQIA protections of data.
- Over 425* radiation oncology facilities across the country are enrolled in the program.
- Over 5,000* safety events have been reported to Clarity PSO.

- Sponsored by:

ASTRO
TARGETING CANCER CARE

AAPM

Supported by:

varian

asrt
American Society of Radiologic Technologists

AAMD • HUMEDIQ • SUN NUCLEAR

*as of March 2018

Why RO-ILS? Why Incident Learning?

- We are dedicated to continuous improvement of quality and safety.
- Increased event reporting (and strong safety culture) is associated with fewer significant adverse events. *Mardon et al. AHRQ, J Patient Saf (2010) 6:226-232*
- Enhance our culture of reporting in a non-punitive learning environment.
- Raise level of awareness about errors and sensitivity to identify error pathways.
- We want to encourage participation from the ENTIRE radiation oncology team including front office, nursing, physicians, dosimetry, physics, and therapy.
- Take advantage of a more user-friendly and time-efficient system.
- Take advantage of learning opportunities from the national database and from our own report analysis using this system.

RO-ILS Benefits

In a recent RO-ILS Participant Survey, enrolled practices have indicated improvement in the following areas due to RO-ILS participation.

- Improved Communication
- Changes to Existing Policies/Workflows
- New Policies/Workflows
- Increase Monitoring of Problem Areas
- Allocation of Resources/Staff

RO-ILS Personnel

- _______ Radiation Oncology department has contracted with Clarity PSO to participate in RO-ILS.

- RO-ILS PSO Liaison
  - Primary contact for Clarity PSO and is responsible for overall participation in RO-ILS by all facilities covered under the contract.
  - Our RO-ILS PSO Liaison is: ____________________

- RO-ILS Reviewers
  - Internal staff who receive event notifications, review, and enter additional analysis information into RO-ILS.
  - Our RO-ILS Reviewers are:
    - ____________________

RO-ILS PROCESS

Incident Learning Cycle

Investigate Event

-Reviewers investigate that event and enter additional information into RO-ILS (i.e., "My Review") .

-Report Event to the PSO.

Review Trends

-Local: Utilizing the Analysis Wizard in the RO-ILS Portal, reviewers analyze trends.

-National: Read RO-ILS Aggregate Reports and additional education.

Mitigation Strategies

•-With multidisciplinary staff, develop mitigation strategies to address issues.

•-Proactively assess processes and identify areas for quality improvement (QI).

Report Findings to All Staff

-Discuss case studies.

-Present national (e.g., Aggregate Report slide deck) and local findings at staff meetings.

-Engage all staff in incident learning and QI implementation.

Submit Event

- User enters an event into RO-ILS (i.e., "Submit Event") upon discovery.

Steps to Submit an Event

1. Access the RO-ILS Portal
    - Click on the RO-ILS Portal Icon on any computer desktop.

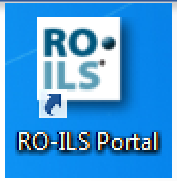

2. Sign in using the generic "Submitter" account information.

    Username: ______________

    Password: ________________

- The shared "Submitter" account allows for anonymous reporting.
- Please do NOT click on "Change Password".
- If you forget the password, please contact

  ____
- The password can also be found on the RO-ILS info sheet

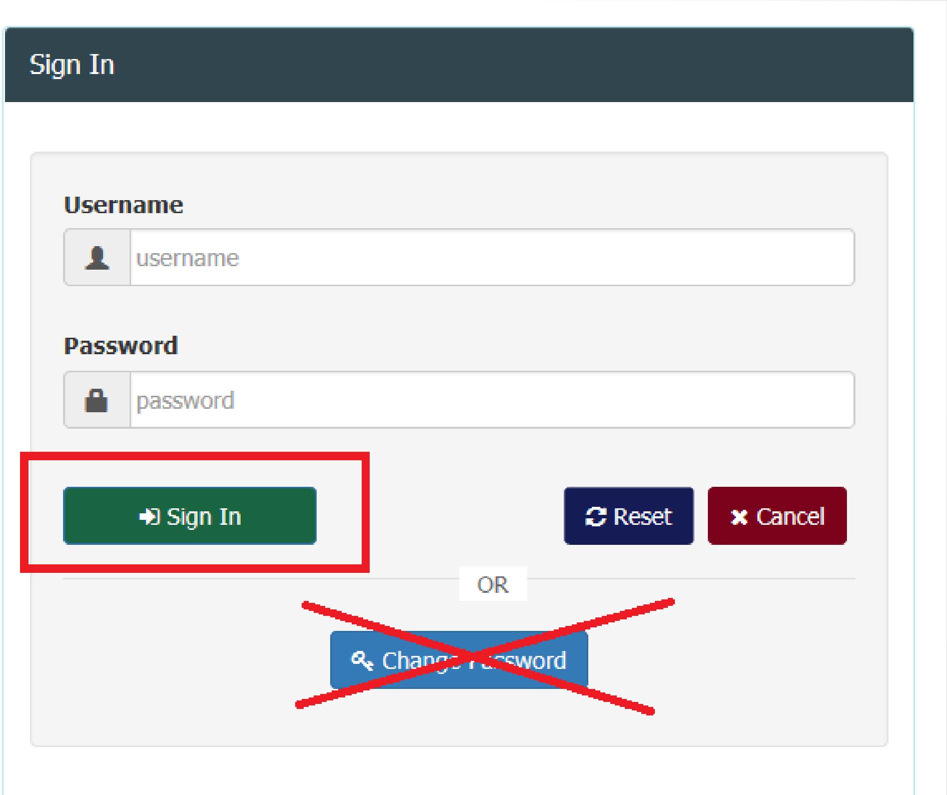

Steps to Submit an Event (cont.)

3. Click on the “Submit Event” button on the very middle of the screen.

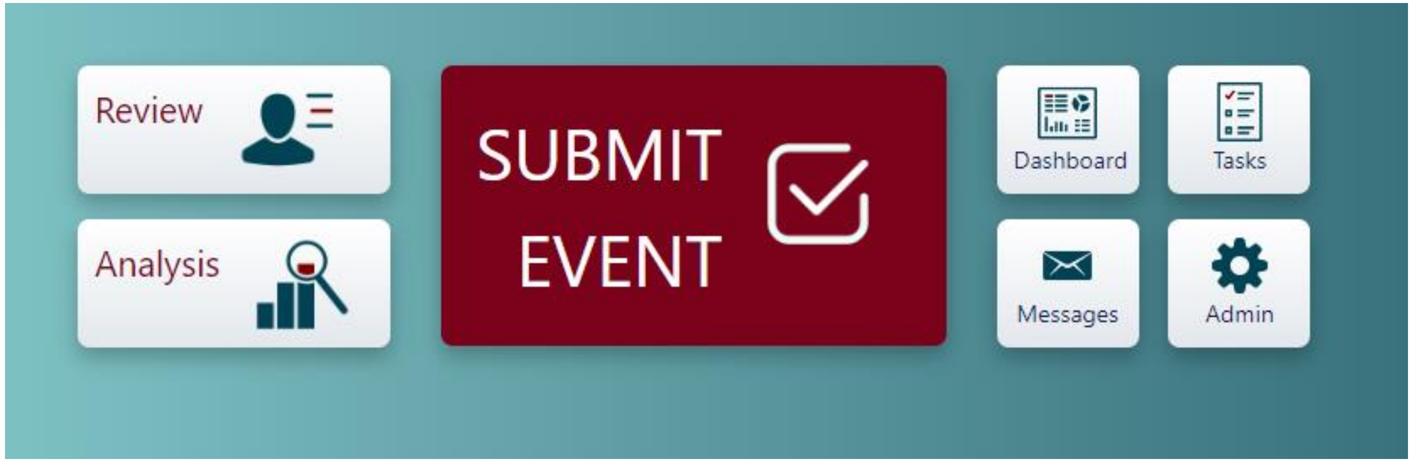

4. Click on the “Event Form” button on the left.

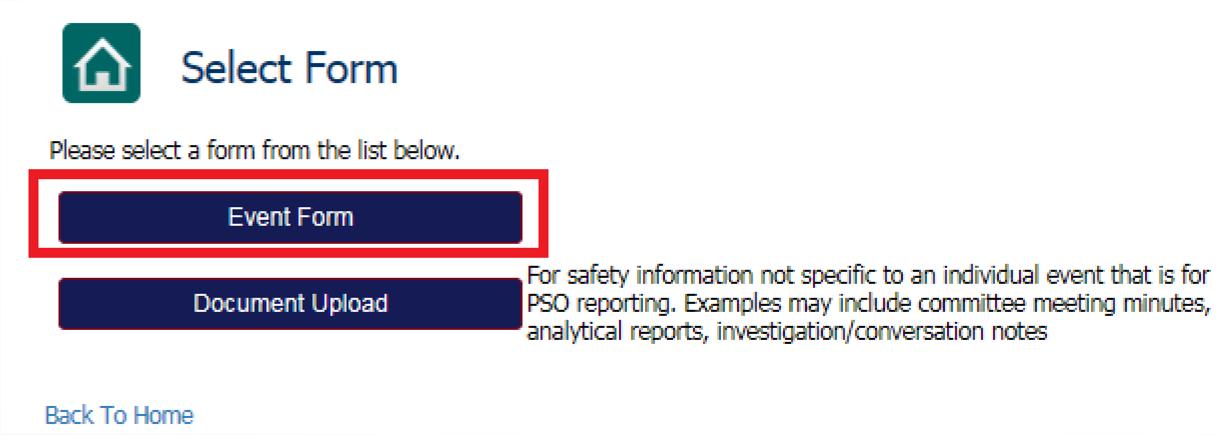

Steps to Submit an Event (cont.)

5. Answer less than 10 data elements and click "Save".

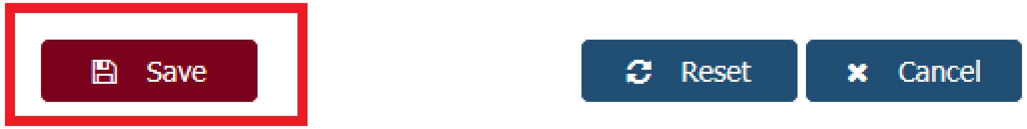

6. Upload any attachments, if available and necessary.

Examples of attachments include:

- Imaging
- Treatment plans
- Dosimetry

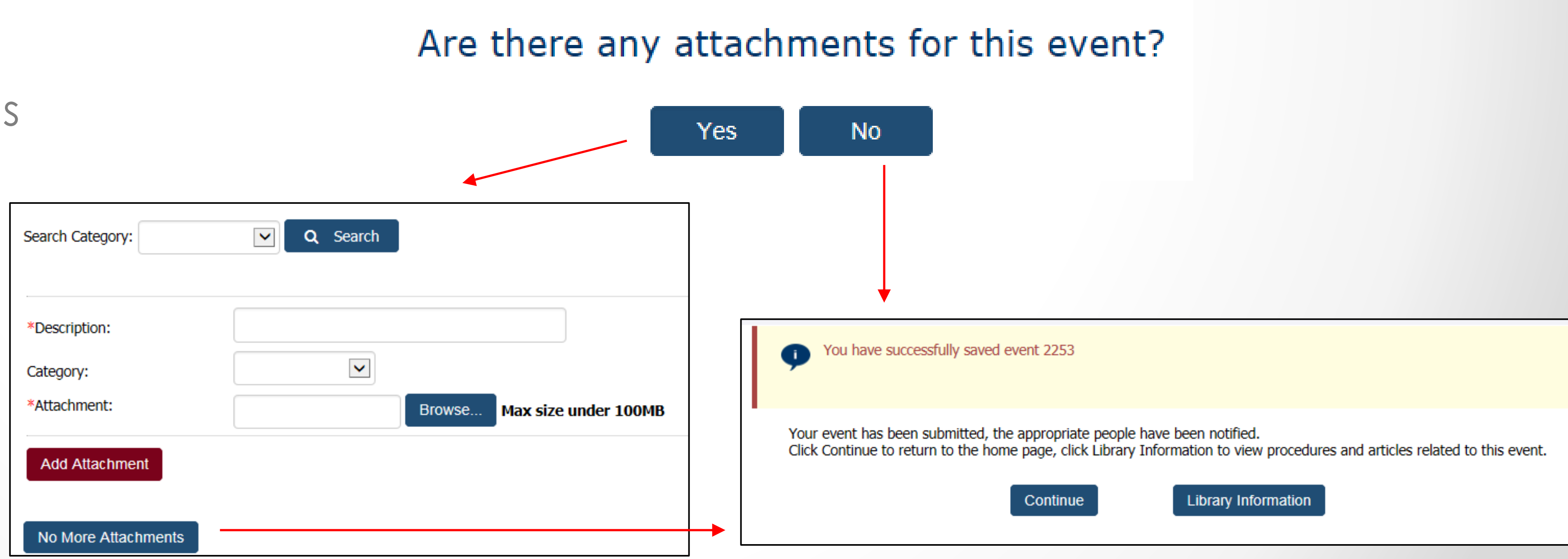

Internal Review Process

- Upon event submission, the reviewers will be notified via email
- If you provide your name, we may contact you for more information
- Reports will be reviewed in _______________
- ______________will decide which events are reported to the PSO
- Only reported events become part of the RO-ILS aggregated data

External Review Process

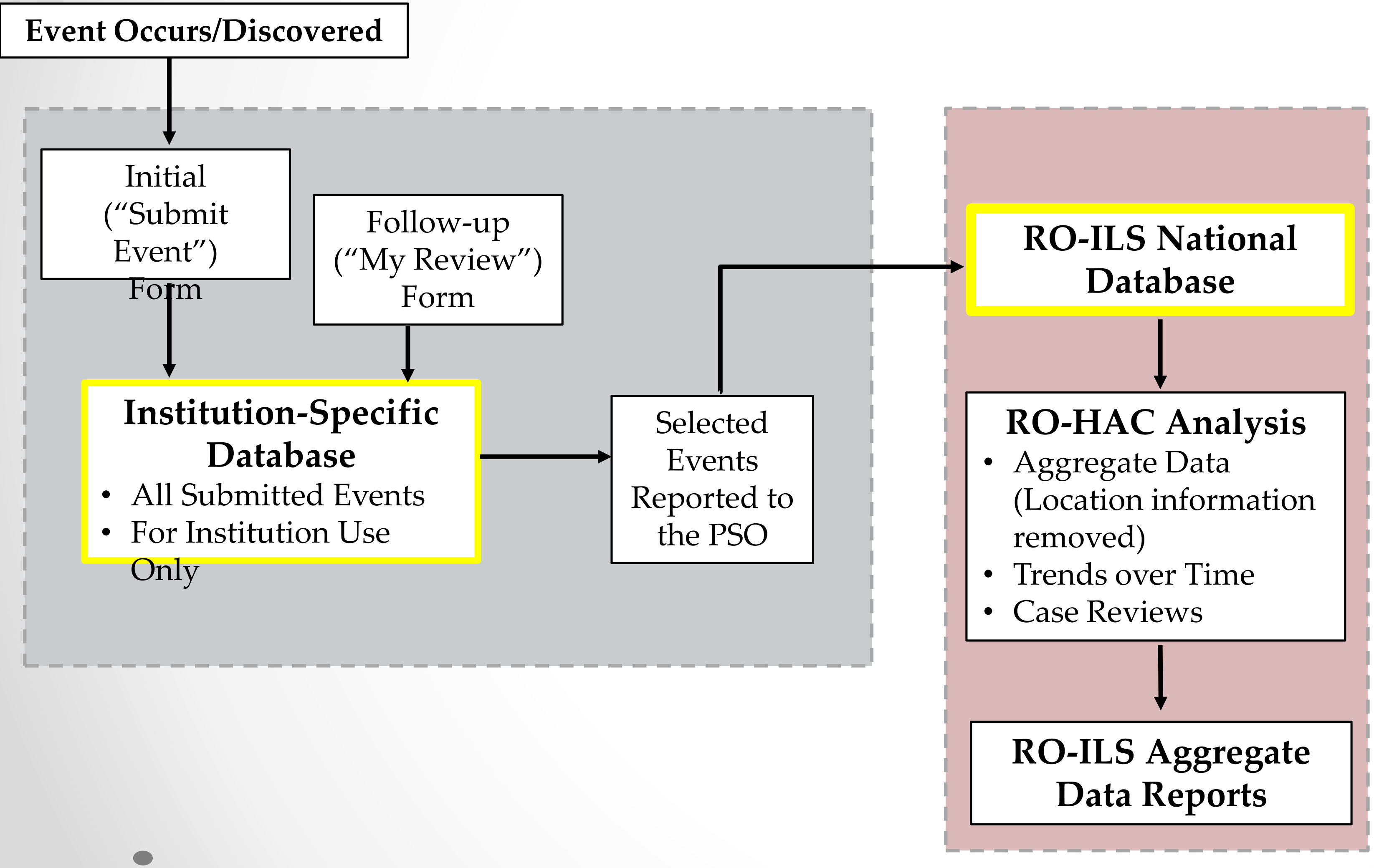

RO-HAC:

- Group of 12 radiation oncologist experts:
  - Radiation oncologists, Physicists, Therapists, a Dosimetrist, Administrator, and Process engineer
- Blindly review ALL events reported to the PSO.
- Develop reports and education based on aggregate findings.

RO-ILS Aggregate Education

- RO-ILS aggregate data is disseminated in regular data reports:
- Reports include a summary report card, analysis & commentary, and graphs.
- Continuing Medical Education (CME) is available for physicians. Continuing education credits also available:
    - 2.00 AMA PRA Category 1 Credit™
    - 2.00 Certificate of Attendance

Below, please find the recent aggregate reports and associated CME:

- Second Quarter 2017 (April 1 - June 30, 2017).......................................*CME Coming Soon!*
- First Quarter 2017 (January 1 - March 31, 2017)..CME
- Fourth Quarter 2016 (October 1 - December 31, 2016)..................................CME
- Third Quarter 2016 (July 1 - September 30, 2016)..CME
- Second Quarter 2016 (April 1 - June 30, 2016)
- First Quarter 2016 (January 1 - March 31, 2016)
- Fourth Quarter 2015 (October 1 - December 31, 2015)
- Third Quarter 2015 (July 1 - September 30, 2015)
- Second Quarter 2015 (April 1 - June 30, 2015)
- First Quarter 2015 (January 1 - March 31, 2015)
- Fourth Quarter 2014 (October 1 - December 31, 2014)
- Third Quarter 2014 (July 1 - September 30, 2014)

Participating practices receive bi-annual reports with practice-level data.

ANALYSIS & COMMENTARY

INTRODUCTION

This quarterly report contains case studies derived from events submitted to RO-ILS: Radiation Oncology Incident Learning System® during the second quarter 2017. The first section identifies an incident with possible medical impact while this quarter's featured theme delves into process improvement (PI): how to learn the most from events and make sustainable changes within your facility. Each of these sections contain interconnected focus topics that highlight an overall theme of learning and improvement of patient safety and quality within radiation oncology through the use of RO-ILS.

HIGH-LEVEL OVERVIEW

At a glance, when comparing data from Q2 2017 to aggregate data from prior quarters (since inception of RO-ILS) there are a number of notable observations. The number of incidents reported over the past several quarter are similar (since Q1 2016), suggesting stable participation and buy-in from RO-ILS participants. However, as the relative stability of quarterly reports in light of increasing number of participants overtime

Report Findings to All Staff

- Trends and mitigation strategies will be disseminated to all staff via ____________________

RO-ILS “SUBMIT EVENT” DATA ELEMENTS

We will use the following 7 slides as we walk through a live submission of a RO-ILS event

Location Data Elements

#101. “Location” is our practice:

- Tufts Medical Center

#102. “Sub location” : N/A

#103. “Additional Location”: N/A

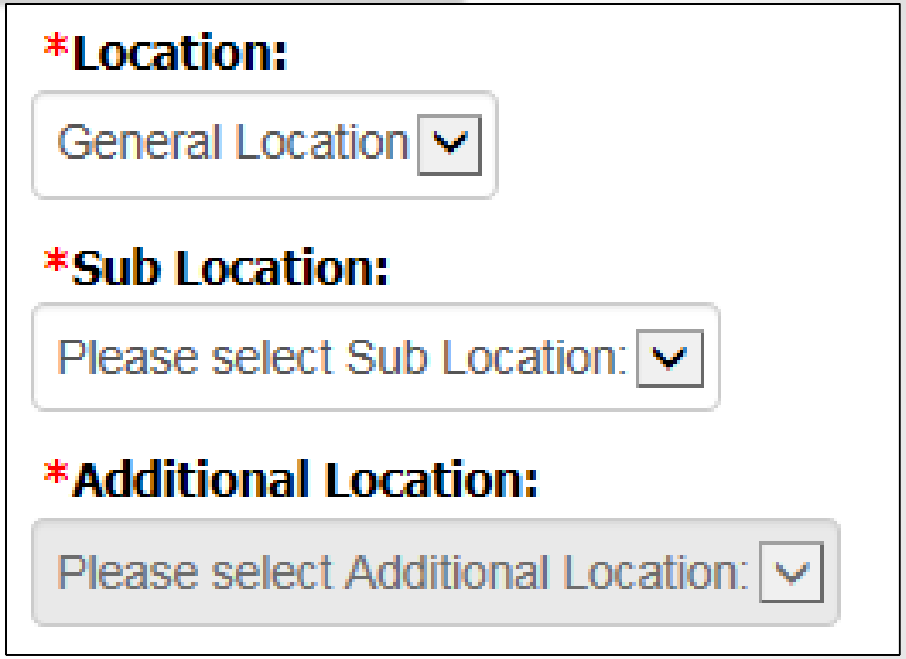

#104. "Event Classification"

Event Classification:

Therapeutic Radiation Incident: Radiation dose not delivered as intended, with or without harm
Other Safety Incident: Event that reached the patient, not involving radiation dose, with or without harm (examples: collision, fall, etc.)
Near-miss: A safety event that did not reach the patient
Unsafe condition: Any condition that increases the probability of a safety event
Operational/Process Improvement: non-safety event

*Event Classification:

Classification Option	Definition	Examples
**Therapeutic Radiation Incident:** Radiation dose delivered not as intended, with or without harm	An event involving the delivery of at least some part of a therapeutic dose. The threshold for this type of event is that a therapeutic field has been initiated.	
**Other Safety Incident:** Event that reached the patient, not involving radiation dose, with or without harm	An event in which a patient is directly affected with or without harm, but does not include some part of a therapeutic dose.	• Collisions with equipment.<br>• Patient falls.<br>• Medication errors.

#104. “Event Classification”

Classification Option	Definition	Examples
**Near-miss:** A safety event that did not reach the patient	An event that relates to the treatment of a specific patient, but does not involve the incorrect delivery of any therapeutic dose.	• A problem discovered by pre-treatment imaging and resolved before any treatment was started (even though some radiation dose was used for the imaging.).<br>• An inconsistency in the documentation for a specific patient, such as a reference in a simulation note to the left side when the right side is being treated.
**Unsafe condition:** Any condition that increases the probability of a safety event	A situation that has potential to affect patients, but not related to a specific patient.	• Noisy environment, chronic haste, lack of documented procedures.<br>• A report from a therapist who is concerned about often being left to treat alone during certain times.
**Operational/Process Improvement:** non-safety event	An event that is not directly related to safety but contributes to inefficiency or confusion; any suggestion to improve department function.	• Problems in scheduling that does not impact patient safety.

#105. “Narrative”

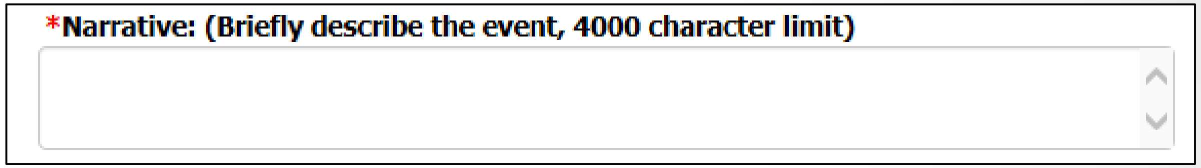

- Briefly tell the story of the event:
  - What happened?
  - How did it happen?
  - Why did it happen? What were the contributing factors?
  - How was it identified?
  - How might it have been prevented? What was the response to the event?
  - What was the level of importance (impact on the patient, if applicable)?
- You may not know the answers to all these questions when submitting an event, but do your best to provide all the relevant information for internal staff (and eventually RO-HAC) review.
- Do NOT include names but DO include roles:
  - E.g., therapist A, planner, MD, etc.

#105. "Narrative"

- Examples of incomplete narrative:
  - Lateral setup port film did not match the digital reconstructed radiograph (DRR). The DRRs were created with the wrong isocenter.
- Example of complete narrative:
  - Patient received one treatment to the thigh that was 5 cm off in the superior-inferior (sup-inf) direction. Image guidance was being done with CBCT. The MD reviewed and approved the images after treatment. During the weekly physics check, the physicist noticed a big change in couch position and reviewed the images. The team agreed that the CBCT of the extremity was not good for localizing in the sup-inf direction and changed to orthogonal X-rays with a larger field of view. A policy was created to require additional review when large shifts are called for.

KEY NARRATIVE COMPONENTS

What happened? How did it happen? Why did it happen? How was it identified? Prevention ideas/Response to event? What was the level of importance?

#106. Treatment Technique

- Select the treatment technique(s) related to the event.
- If this information is not relevant for the report (e.g., slippery hallway floor – an unsafe condition), then select "Not Applicable".
- If "LDR", "HDR", or "Radiopharmaceuticals" options are selected a follow-up question will appear and the radioisotope and manufacturer should be entered.
- If "Other" selected, please specify.

***Treatment Technique Pertinent to Event: (Select all that apply)**

☐ 2D	☐ Electrons	☐ Radiopharmaceuticals
☐ 3D	☐ Intraoperative	☐ Total body irradiation (TBI)
☐ IMRT/VMAT	☐ kV x-rays (i.e. Orthovoltage and superficial)	☐ Not Applicable
☐ SRS/SBRT	☐ LDR	☐ Other
☐ Particles (Protons)	☐ HDR	

List LDR radioisotope, model, temporary/permanent, and manufacturer:

List HDR radioisotope and delivery unit manufacturer and model:

List the radioisotope type and manufacturer:

***Specify 'Other' Treatment Technique:**

Data Elements

#107 Local Identifier:

#108 Reporter's Name:

- We encourage you provide your name so we can better investigate the event but this is *not required*.
- There will be no reprisal for reporting an event.

#109 Date and Time the Event Occurred:

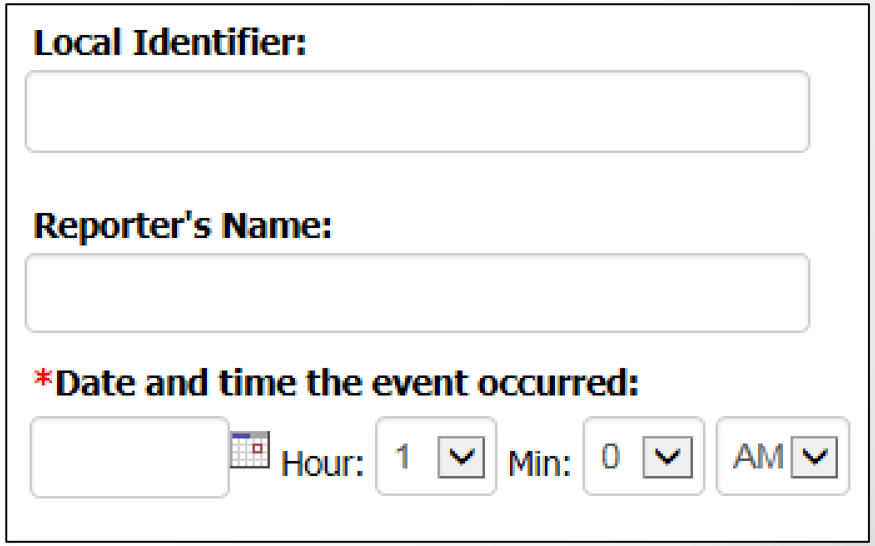

RO-ILS FOLLOW-UP EXAMPLE

We will now walk through a live follow-up

Considerations

To encourage a positive environment please consider the following:

- Plan ahead: coordinate with the person you would like to follow-up with and find a time that is good for both parties to discuss. It would be beneficial to provide the person with enough information to re-familiarize them with the event you would like to discuss.

- Be conscience of other tasks: Please try to avoid discussing events while the reporter is doing other tasks. Distraction generally does not help with patient safety so let's try to minimize it!

- Be timely: Follow-ups are much easier for all parties if they are done in close proximity to when the events occurred. I will be working on trying to get events assigned faster as well to help aid in this

Root Cause Analysis

- Method of problem solving used for identifying the root causes of faults or problems
- Basic or root causes: generically describes conditions which predispose a system to failure.
  - Should be interpreted to include contributing factors and latent conditions which increase the probability of system failure but don't make it inevitable
- Note: both incidents and near-misses can be analyzed
  - Typically RCA is primarily done on incidents
- Important to do the RCA as close to the occurrence of the event
- RCA should lead to implementation of new workflow and communication methods

RCA

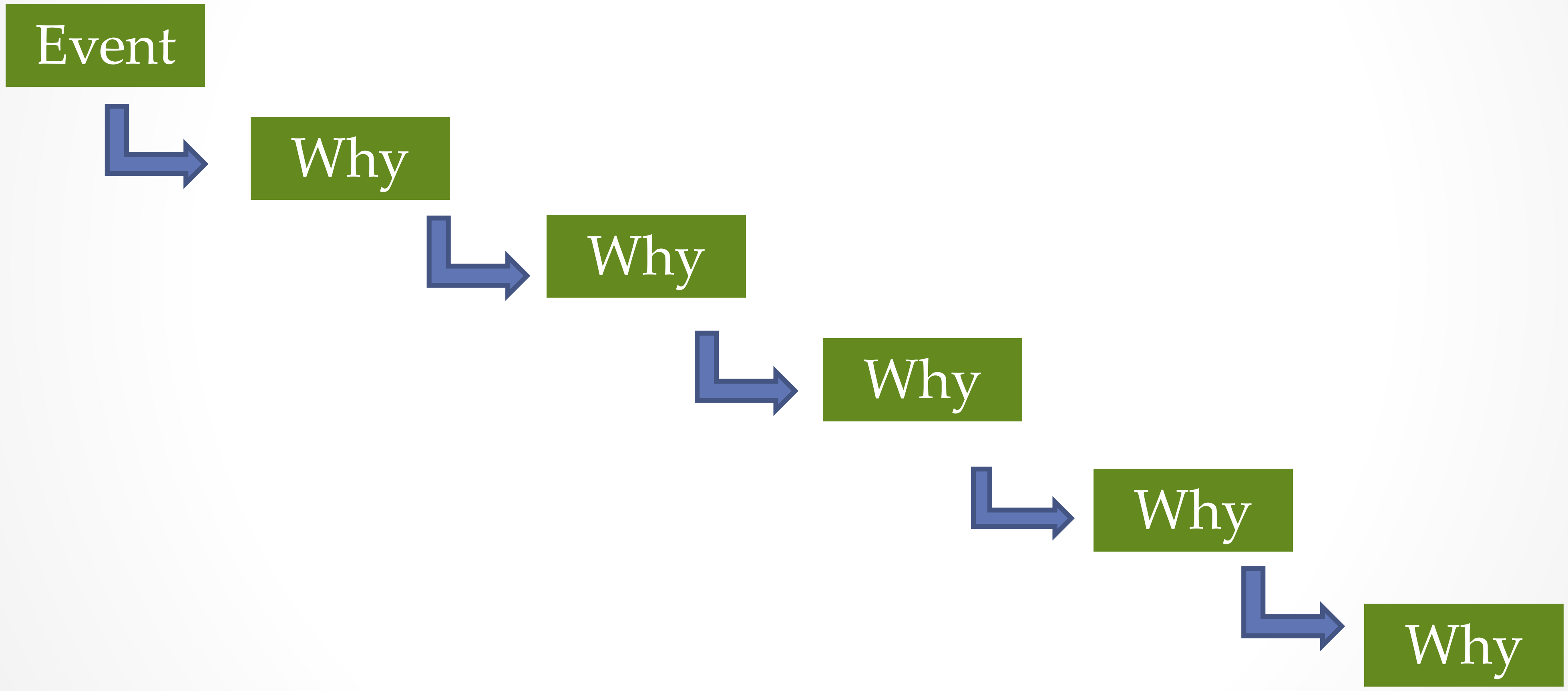

Root Cause Analysis

- Step 1: Prepare a detailed, chronological description of events that led up to the incident
- Step 2: Trace through review of relevant clinical documents and interviews of involved persons, the basic or root causes of the incident (the 5 why's)
- Step 3: Establish the relationships between the incident and basic or root causes (cause and effect diagram)
- Steps 3+: Develop preventative actions. A learning organization will disseminate lessons learnt from RCA

Root Cause Analysis

- Experience shows that many of the basic causes identified in connection with one incident are issues across the whole clinical program
- The benefit of RCA extends beyond minimizing the probability of one or a few particular incidents being repeated

Failure Modes and Effects Analysis

- FMEA is a risk assessment tool used to identify weakness or deficiencies in process
- A step by step approach for assessing postulated failure modes in a clinical process
- FMEA helps us prioritize postulated failure modes for further analysis
- **"Failure modes"** means the ways, or modes, in which something might fail. Failures are any errors or defects, especially ones that affect patients, and can be potential or actual
- **"Effects analysis"** refers to studying the consequences of those failures.

Failure Modes and Effects Analysis

FMEA document general format / TABLE 1

May be a product, assembly, subassembly or part

Initial development of the FMEA									Improvement activities		Post-improvement activities				
Process step/ input	Potential failure mode	Potential failure effects	SEV	Potential causes	OCC	Current controls	DET	RPN	Actions recom-mended	Resp.	Actions taken	SEV	OCC	DET	RPN
1	2	3	4	5	6	7	8	9	10	11	12	13			

DET = detection
FMEA = failure mode and effects analysis
OCC = occurence
Resp = responsible
RPN = risk priority number
SEV = severity

Questions?
