# Supplementary material for: Implementation of a patient safety training program in radiation oncology residency: A pilot study
Source: J Appl Clin Med Phys. 2024 Feb 15;25(3):e14286. doi: 10.1002/acm2.14286 (PMC10929992; doi:10.1002/acm2.14286)
Supplement: Supplementary file 3 — Supporting Information [file ACM2-25-e14286-s002.docx]

Supplemental 3

**Incident Learning Education Post- Residency Survey**

Please indicate your satisfaction with the RO-ILS based Incident Learning Education Program

1. Did you participate in the RO-ILS Education Program while rotating at our institution during your residency?
   1. Yes
   2. No
2. If you did NOT participate in the RO-ILS education program, would more instruction during residency have been helpful to you based on your experience post residency?
   1. Yes
   2. No
   3. Maybe
   4. N/A
3. Does your current institution participate in any incident learning program?
   1. Yes
   2. No
4. If your institution participates, is the incident learning program specific to radiation oncology?
   1. Yes
   2. No
   3. N/A
5. If your institution participates, did you have any influence in the decision to participate in an incident learning program?
   1. Yes
   2. No
   3. N/A
6. If your institution does NOT participate in incident learning, do you have plans to help implement a program in the future?
   1. Yes, and I feel that I have the time, resources, and support to do so
   2. No, and I do not have interest or a need to have an incident learning program at my institution
   3. I would like to, but I do not feel as though I have institutional support
   4. I would like to, but I do not have the time resources to dedicate to this
   5. N/A

**Please answer the following questions if you participated in the RO-ILS Education Program**

1. How valuable was the RO-ILS education program to your board preparation?

Not Valuable 1 2 3 4 Very Valuable

1. How valuable was the RO-ILS education program to your current role?

Not Valuable 1 2 3 4 Very Valuable

1. How valuable do you feel the RO-ILS education program was to your general knowledge?

Not Valuable 1 2 3 4 Very Valuable

1. Have you utilized your RO-ILS education program knowledge post residency?

Not At All 1 2 3 4 Very Much

1. How likely are you to use your patient safety knowledge gained by the RO-ILS education program in the future?

Not Very Likely 1 2 3 4 Very Likely

1. Do you have any other feedback or comments to provide to the program?

______________________________________________________________
